# Characterization of *Salmonella* Typhimurium isolates from domestically acquired infections in Finland by phage typing, antimicrobial susceptibility testing, PFGE and MLVA

**DOI:** 10.1186/s12866-015-0467-8

**Published:** 2015-07-02

**Authors:** Taru Lienemann, Aino Kyyhkynen, Jani Halkilahti, Kaisa Haukka, Anja Siitonen

**Affiliations:** Bacterial Infections Unit, National Institute for Health and Welfare, P.O. BOX 30, 00271 Helsinki, Finland; Department of Food and Environmental Sciences, Division of Microbiology, University of Helsinki, P.O. BOX 56, 00014 Helsinki, Finland

**Keywords:** *Salmonella* Typhimurium, Molecular subtyping, PFGE, MLVA, Phage typing, Antimicrobial resistance profiling, MDR

## Abstract

**Background:**

*Salmonella enterica* spp. *enterica* serotype Typhimurium (STM) is the most common agent of domestically acquired salmonellosis in Finland. Subtyping methods which allow the characterization of STM are essential for effective laboratory-based STM surveillance and for recognition of outbreaks. This study describes the diversity of Finnish STM isolates using phage typing, antimicrobial susceptible testing, pulsed-field gel electrophoresis (PFGE) and multilocus variable-number tandem repeat analysis (MLVA), and compares the discriminatory power and the concordance of these methods.

**Results:**

A total of 375 sporadic STM isolates were analysed. The isolates were divided into 31 definite phage (DT) types, dominated by DT1 (47 % of the isolates), U277 (9 % of the isolates) and DT104 (8 % of the isolates). Of all the isolates, 62 % were susceptible to all the 12 antimicrobials tested and 11 % were multidrug resistant. Subtyping resulted in 83 different XbaI-PFGE profiles and 111 MLVA types. The three most common XbaI-PFGE profiles (STYM1, STYM7 and STYM8) and one MLVA profile with three single locus variants accounted for 56 % and 49 % of the STM isolates, respectively. The studied isolates showed a genetic similarity of more than 70 % by XbaI-PFGE. In MLVA, 71 % of the isolates lacked STTR6 and 77 % missed STTR10p loci. Nevertheless, the calculated Simpson’s diversity index for XbaI-PFGE was 0.829 (95 % CI 0.792−0.865) and for MLVA 0.867 (95 % CI 0.835−0.898). However, the discriminatory power of the 5-loci MLVA varied among the phage types. The highest concordance of the results was found between XbaI-PFGE and phage typing (adjusted Wallace coefficient was 0.833 and adjusted Rand coefficient was 0.627).

**Conclusions:**

In general, the calculated discriminatory power was higher for genotyping methods (MLVA and XbaI-PFGE) than for phenotyping methods (phage typing). Overall, comparable diversity indices were calculated for PFGE and MLVA (both DI > 0.8). However, MLVA was phage type dependent providing better discrimination of the most common phage types. Furthermore, 5-loci MLVA was a less laborious method and easier to interpret than XbaI-PFGE. Thus, the laboratory-based surveillance of the Finnish human STM infections has been conducted with a combination of phage typing, antimicrobial susceptibility testing and 5-loci MLVA since January 2014.

## Background

By a rough estimation, 5.1 million foodborne non-typhoidal salmonella infections and consequently 8400 deaths occur in Europe annually [1]. *Salmonella enterica* ssp. *enterica* serotype Typhimurium (STM) is, after the serotype Enteritidis, the second most commonly isolated serotype from human salmonelloses in Europe [2]. In Finland, the total number of reported salmonella infections ranged from approximately 2200 to 3100 cases per year in 2007–2012 [3]. Of these infections, 10–30 % were considered domestically acquired and serotype Typhimurium dominated among the domestic isolates [3]. Most salmonelloses are characterized by mild-to-moderate self-limited diarrhoea but also serious disease resulting in death has been reported especially in connection with STM infection in children less than 5 years old [4, 5]. Salmonella infections, including those caused by STM, are considered sporadic and their sources remain unknown. However, national outbreaks [6, 7] and large outbreaks across the national borders associated with STM have also been reported [8, 9]. The STM outbreaks are frequently associated with consumption of contaminated raw vegetables, fruits, poorly cooked meat or meat products and eggs [10–13].

Molecular subtyping of bacterial isolates is crucial when monitoring the circulation of bacterial strains across the geographic regions. Subtyping methods must fulfil many requirements such as high discriminatory power, easy performance, clear interpretation and possibility for international standardization. Currently, pulsed-field gel electrophoresis (PFGE), which has been internationally standardized, is considered as the golden standard for genotyping of *Salmonella* and it is the only generic molecular method suitable for all *Salmonella* serotypes [14–16]. However, not all the isolates unrelated to an outbreak are different by PFGE from the outbreak-related isolates and PFGE has been shown to suffer from a high variation between the different laboratories. Thus, new typing methods have been developed around the world. Among these, multilocus variable-number tandem repeat analysis (MLVA) has been used as a typing tool for tracing back the sources of foodborne disease outbreaks, in bacterial surveillance and in research [17]. MLVA can be used to discriminate between genetically closely related strains [18]. A database of tandem repeats for several completed *Salmonella* genome sequences is publicly available at http://mlva.u-psud.fr/mlvav4/genotyping/ [19]. These variable-number tandem repeat (VNTR) markers have been applied for typing and clustering of several *Salmonella* serotypes, for example, Dublin [20], Enteritidis [21], Gallinarum [22], Montevideo [23], Newport [24], Paratyphi A [25], Typhi [26] and Typhimurium [27]. MLVA typing has also been used successfully in several STM outbreak investigations [28–30]. Reduced typing time, easy handling and clearer interpretation of MLVA results, compared to those of PFGE, are beneficial in an outbreak situation. The major disadvantage of MLVA is serotype-specificity. There is, for example, no MLVA protocol which is suitable for all the over 2600 *Salmonella* serotypes and, currently, internationally standardized MLVA protocols are available for only a few serotypes. In Europe, the 5-loci MLVA scheme developed by Lindstedt *et al.* 2004, has been widely adopted for STM by several national public health laboratories [17]. The MLVA loci nomenclature devised by Larson *et al.*, 2009 allows the normalisation of the raw data and enables a direct comparison of the MLVA results between laboratories [31].

The aim of this study was to characterize Finnish STM isolates using several pheno- and genotyping methods. For that, 375 sporadic STM isolates from Finns who had not been abroad recently were characterized by phage typing, antimicrobial susceptibility testing, XbaI-PFGE and 5-loci MLVA during the 5-year period between November 2007 and October 2012. The specific aim was to determinate (i) the genetic diversity among the Finnish STM isolates, especially those of endemic definitive phage type DT1, and to detect clonal clusters, (ii) to compare the discriminatory power of XbaI-PFGE and 5-loci MLVA and (iii) to investigate whether the 5-loci MLVA results are in concordance with the phage typing or XbaI-PFGE results. In addition, the potential and possible added value of 5-loci MLVA as a typing tool in a laboratory for the STM surveillance was evaluated.

## Results

### Overall diversity of the isolates

The 375 STM isolates from Finns, who had not travelled abroad recently were typed by phage typing, antimicrobial susceptibility testing, PFGE and MLVA (5 primer pairs in Table 1). Among the isolates, 31 distinct phage types were detected (Table 2). Of the isolates, 65 % (245 isolates) were assigned to the three most common DTs: DT1 (48 % of the isolates), U277 (9 % of the isolates) and DT104 (8 % of the isolates). Each of the remaining phage types contained less than 4 % of the isolates (Table 2). The isolates associated with the phage type called “reacts but does not conform” (RDNC) accounted for 10 % (38 isolates). Based on similar phage reactions, these isolates were divided into five groups RDNC1-5 (22 isolates). In addition, 16 isolates showing each a unique RDNC pattern were found. Of all the isolates, 62 % (231 isolates) were susceptible to all antimicrobial agents tested. Within the most common phage types, 74 % (133 of 180isolates) of DT1, 87 % (29 of 33) of U277 but only 3 % (1 of 32) of DT104 isolates was susceptible to all antimicrobials tested. On the whole, 11 % (43 isolates) were multidrug resistant (MDR) (Table 2). The three most common MDR patterns were ACSSuTNx (14 isolates), ACSSuT (10 isolates) and ASSuT (7 isolates), representing 8 % of the isolates. Of the DT104 isolates with any resistance marker, 45 % (14 isolates) displayed resistance to six antimicrobials (ACSSuTNx). All of the 14 Nx resistant isolates had a decreased sensitivity to ciprofloxacin (MIC values were ranging from 0.125 to 0.19 mg/L). In addition to the 14 DT104isolates, a decreased sensitivity to ciprofloxacin was observed in two DT120 (0.25–0.38 mg/L), two U311 (0.19–0.38 mg/L) and one DT104B (3 mg/L) isolates. Furthermore, one isolate of phage type U311 showed combined resistance to more than six antimicrobials including an intermediate resistance to cefotaxime. None of the isolates were resistant to imipenem.

**Table 1:** Characteristics of each MLVA loci for *S*. Typhimurium

Locus	Repeat length (bp)	Reference strain (location)	Primer sequence (3’ → 5’)	Smallest product (bp)	Largest product (bp)	Gene
STTR3	27/33	LT2 (3629458)	STTR3-F:NED-ccccctaagcccgataatgg	464	530	*bigA*
			STTR3-R: tgacgccgttgctgaaggtaataa			
STTR5	6	LT2 (3184543)	STTR5-F:NED-atggcgaggcgagcagcagt	228	300	*yohM*
			STTR5-R:ggtcaggccgaatagcaggat			
STTR6	9	LT2 (2730867)	STTR6-F:6FAM-tcgggcatgcgttgaaa	283	397	Prophage related
			STTR6-R: ctggtggggagaatgactgg			
STTR9	9	LT2 (3246672)	STTR9-F:6FAMagaggcgctgcgattgacgata	163	181	Intergenic
			STTR9-R: cattttccacagcggcagtttttc			
STTR10p	6	pSLT (53711)	STTR10-F: PETcgggcgcggctggagtatttg	358	496	In plasmid
			STTR10-R: gaaggggccgggcagagacagc

**Table 2:** Distribution of phage types, multidrug resistance, PFGE profiles and MLVA types among the 375 STM isolates from the domestic infections

Phage type	No. Isolates ( % of total)	MDR^a^ (No. of isolates)	No. PFGE profiles within one phage type	The most common PFGE profiles (number of strains, %)	No. MLVA profiles within one phage type	The most common MLVA profiles (number of strains, %)
DT1	180 (48 %)	ASSuTm (1)	23	STYM1 (140, 77 %)	20	3-16-NA-NA-0311 (105, 58 %)
				STYM100 (5, 3 %)		3-15-NA-NA-0311 (23, 13 %)
				STYM262 (4, 2 %)		3-18-NA-NA-0311 (8, 4 %)
U277	33 (9 %)	-	7	STYM8 (26, 79 %)	7	3-16-NA-NA-0311 (14, 42 %)
				STYM265 (3, 9 %)		3-16-NA-NA-0211 (6, 18 %)
						3-18-NA-NA-0311 (6, 18 %)
DT104	32 (8 %)	ACSSuTNx (14), ACSSuT (3)	7	STYM7 (24, 75 %)	18	3-13-5-12-0311 (11, 34 %)
				STYM47 (3, 9 %)		3-14-16-21-0311 (3, 9 %)
DT116	14 (4 %)	ASuTTm (2), ASSuT (1)	6	STYM243 (6, 42 %)	7	5-14-NA-NA-0611 (6, 42 %)
				STYM275, (3, 21 %)		2-19-NA-NA-0211 (3, 21 %)
DT120	11 (3 %)	ACSSuT (1), SSSuT (1)	7	STYM194 (3, 27 %)	8	3-12-9-NA-0211 (3, 27 %)
				STYM154 (3, 27 %)		2-13-NA-NA-0311 (2, 18 %)
DT41	12 (3 %)	-	5	STYM52 (7, 58 %)	11	3-16-NA-NA-0311 (2, 16 %)
				STYM1 (2, 16 %)		
DT104B	10 (3 %)	ACSSuT (4), ASSuT (4),	5	STYM7 (5, 50 %)	10	10 individual profiles
		ACSSuTTm (1), CSSuTGNx (1)				
RDNC4	9 (2 %)	-	2	STYM42 (8, 89 %)	3	3-17-NA-NA-0311 (7, 78 %)
RDNC2	6 (2 %)	-	2	STYM8 (3, 50 %)	3	3-14-NA-NA-0311 (3, 50 %)
				STYM265 (3, 50 %)		3-15-NA-NA-0311 (2, 33 %)
DT2	6 (2 %)	-	2	STYM141 (5, 83 %)	3	2-14-12-8-0212 (4, 67 %)
DT193	6 (2 %)	ACSSuT (1), ASuTTm (1), ASSuT (1)	6	6 individual profiles	6	6 individual profiles
DT195	5 (1 %)	-	4	STYM8 (2, 40 %)	5	5 individual profiles
DT12	4 (<1 %)	-	3	STYM238 (2, 50 %)	2	4-13-9-7-211 (3, 75 %)
U282	4 (<1 %)		3	STYM42 (2, 50 %)	3	3-16-NA-NA-311 (2, 50 %)
U302	4 (<1 %)	ACSSuT (1)	2	STYM228 (3, 75 %)	2	2-15-NA-NA-211 (3, 75 %)
RDNC1	3 (<1 %)	-	1	STYM124 (3, 100 %)	3	3 individual profiles
DT8	2 (<1 %)	-	2	2 individual profiles	2	2 individual profiles
DT9	2 (<1 %)	-	1	STYM285 (2, 100 %)	2	2 individual profiles
U311	2 (<1 %)	ACSSuTTmNx (1), ASuTNx (1)	2	2 individual profiles	2	2 individual profiles
U312	2 (<1 %)	-	1	STYM119 (2, 100 %)	2	2 individual profiles
RDNC3	2 (<1 %)	-	2	2 individual profiles	2	2 individual profiles
RDNC5	2 (<1 %)	-	1	STYM8 (2, 100 %)	2	2 individual profiles
DT7	1	-	1		1	
DT10	1	-	1		1	
DT15A	1	-	1		1	
DT40	1	-	1		1	
DT99	1	-	1		1	
DT132	1	-	1		1	
DT135	1	-	1		1	
NT	1	-	1		1	
RDNC	16 induvidual phage reactions	ACSuTTm (1), ASSUT (1)	14	STYM1 (2, 14 %)	14	3-16-NA-NA-0311 (2, 14 %)
31 individual phage types	total 375 isolates	43 multiresistant isolates	83 individual PFGE types		115 individual MLVA types	

*DT*, Definite phage type

*RDNC*, Reacts but does not confirm

*NT*, Not typeable

*MDR*, Multidrug resistance, ^a^ only R counted as resistance (I not included)

Antimicrobials: ampicillin (A), chloramphenicol (C), streptomycin (S), sulphonamide (Su), tetracycline (T), trimethoprim (Tm), gentamicin (G), nalidixic acid (Nx)

*PFGE*, Pulsed-field gel electrophoresis

*MLVA*, Multilocus variable-number tandem repeat analysis

*NA*, No amplification

In the molecular analyses, 83 different XbaI-PFGE and 111 different MLVA profiles were generated (Table 2). Among DT1 isolates, the most common XbaI-PFGE profile STYM1 contained 10 MLVA profiles, of which the MLVA profile 3-16-NA-NA-0311 was the most prevalent. The U277 isolates were divided into six XbaI-PFGE and seven MLVA profiles (Table 2). The most common XbaI-PFGE profile STYM8 showed five different MLVA profiles, of which again the 3-16-NA-NA-0311 was the most common. The DT104 isolates were divided into six XbaI-PFGE and 17 MLVA profiles (Table 2). Here, the most common XbaI-PFGE profile STYM7 contained 13 different MLVA profiles, of which the profile 3-13-5-12-0311 was the most common.

Although higher number of MLVA types (n = 111) than XbaI-PFGE profiles (n = 83) were generated (p ≤ 0.05), the calculated diversity indices for MLVA (Simpson’s DI = 0.867 with 95 % CI 0.835−0.898; Shannon’s DI, H’ = 4.697) and for XbaI-PFGE (Simpson’s DI = 0.829 with 95 % CI 0.792−0.865; Shannon’s DI, H’ = 4.207) were comparable and the confidence intervals partly overlapped (Table 3). The discriminatory power of MLVA was better for the three most common phage types compared to XbaI-PFGE. In general, the MLVA data obtained in this study suggest that MLVA better performance compared to XbaI-PFGE varies with phage type (Table 4). In general, the MLVA data that we have obtained suggest that MLVA is phage type dependent. The discriminatory power of MLVA was better for the five most common phage types compared to XbaI-PFGE (Table 4). In addition, the Simpson’s diversity indices for each of the five MLVA loci varied being highest for locus STTR5 (DI = 0.789; 95 % CI 0.780−0.811) and lowest for locus STTR9 (DI = 0.328; 95 % CI 0.298-0.358) (Table 5). The highest variability in repeat numbers at one locus was found in locus STTR6 (21 different repeats) and the lowest in locus STTR9 (7 different repeats) (Table 5).

**Table 3:** Discriminatory power of three typing methods for 375 sporadic *S.* Typhimurium isolates

Method	No. of profiles	Simpson’s DI (95 % CI)	Shannon’s DI (H_min_-H_max_)
PT	31	0.749 (0.703-0.794)	H’ = 3.127 (0.795-4.954), J = 0.631
Xbal-PFGE	83	0.829 (0.792-0.865)	H’ = 4.207 (2.148-6.375), J = 0.660
5-loci MLVA	111	0.867 (0.835-0.898)	H’ = 4.697 (2.862-6.794, J = 0.691

*PT*, Phage typing

*PFGE*, Pulsed-field gel electrophoresis

*MLVA*, Multilocus variable-number tandem repeat analysis

*DI*, Simpson’s diversity index

*95 % CI*, Confidence Interval, precision of the diversity index, expressed as 95 %

upper & lower boundaries

*H*, Indicator of species richness

*J*, Indicator of the evenness of subtype distribution

*H*_*min*_ *-H*_*max*_, Precision of the Shannon’s diversity index (H’)

**Table 4:** Simpson’s diversity indices of Xbal-PFGE and 5-loci MLVA among the five most common phage types

Phage type	DI for Xbal-PFGE (95 % CI)	DI for 5-loci MLVA (95 % CI)
DT1	0.394 (0.299-0.488)	0.637 (0.560-0.714)
U277	0.379 (0.170-0.587)	0.759 (0.659-0.860)
FT104	0.389 (0.177-0.601)	0.875 (0.776-0.974)
DT116	0.791 (0.632-0.950)	0.802 (0.632-0.972
DT120	0.891 (0.782-1.0)	0.927 (0.833-1.0)

*DT*, Definite phage type

*DI*, Simpson’s diversity index

*95 % CI*, Confidence Interval, precision of the diversity index, expressed as 95 %

upper & lower boundaries

**Table 5:** Simpson’s diversity index of each MLVA loci

MLVA locus	DI (95 % CI) for DT1 (n = 180)	DI (95 % CI) for U277 (n = 33)	DI (95 % CI) for DT 104 (n = 32)	DI (95 % CI) for all DTs (n = 375)	k-value for all DTs
STTR9	0.065 (0.015-0.115)	0.000 (0.000-0.189)	0.119 (0.000-0.271)	0.328 (0.298-0.358)	7
STTR5	0.574 (0.499-0.648)	0.571 (0.418-0.724)	0.742 0.652-0.833)	0.795 (0.780-0.811)	19
STTR6	0.097 (0.037-0.157)	0.000 (0.000-0.189)	0.807 (0.706-0.908)	0.494 (0.462-0.527)	21
STTR10	0.076 (0.022-0.129)	0.059 (0.000-0.169)	0.752 (0.655-0.849)	0.408 (0.375-0.441)	20
STTR3	0.211 (0.135-0.288)	0.298 (0.130-0465)	0.225 (0.042-0.407)	0.438 (0.409-0.467)	10

*DI*, Simpson’s diversity index

*95 % CI*, Confidence Interval, precision of the diversity index, expressed as 95 %

upper & lower boundaries

*DT*, Definite phage type

*k-value*, Number of different repeats present at each locus in this sample set

### Relatedness of the sporadic isolates by PFGE and MLVA

**Fig. 1:**
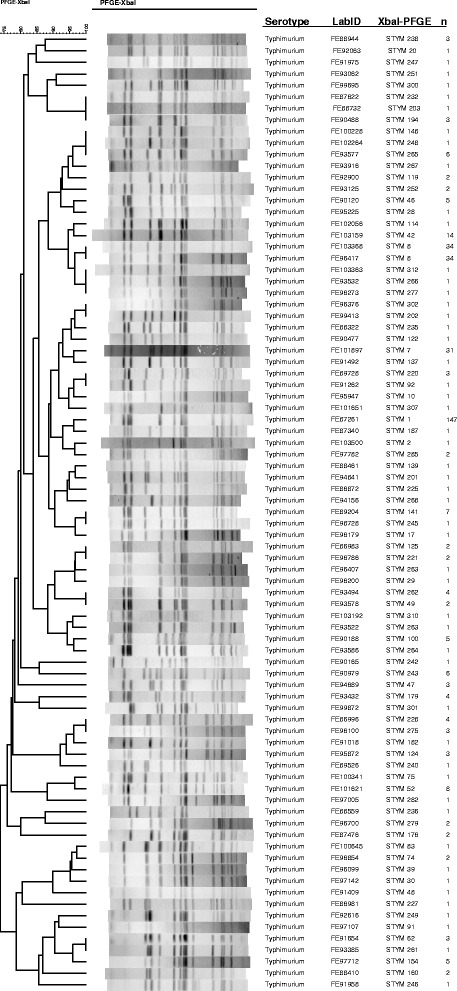
A UPGMA dendrogram of XbaI-PFGE pattern of 375 STM isolates. Each XbaI-PFGE profile (n = 83) is presented only once and the number of isolates column provides information on frequency of each XbaI-PFGE profile. STYM1 (n = 147) was the most common profile and 50 isolates had a unique profile

**Fig. 2:**
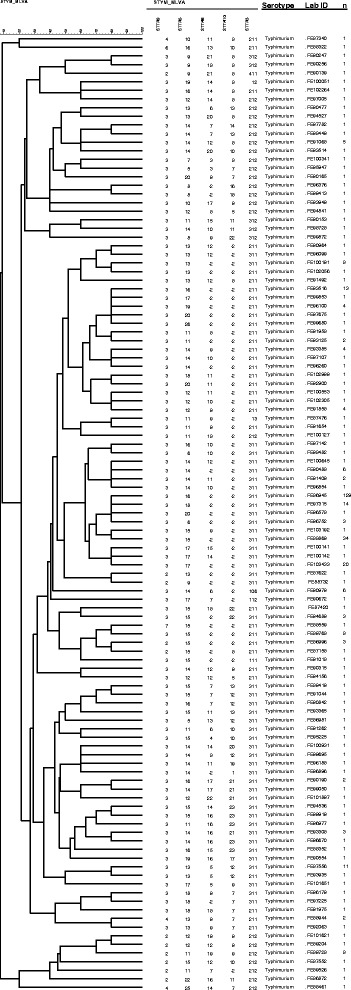
A UPGMA dendrogram of the MLVA types of 375 STM isolates. Each MLVA type (n = 111) is presented only once and the number of isolates column provides information on frequency of each MLVA type. MLVA type 3-16-NA-NA-0311 (n = 129) was the most common and 89 isolates had a unique MLVA type

UPGMA clustering was done by construction of dendrograms for both PFGE and MLVA profiles (Figs. 1 and 2). The cluster analysis of XbaI-PFGE results demonstrated a genetic similarity of more than 70 % among the studied isolates (Fig 1). In 11 cases the PFGE banding patterns differed from each other only by the thickness of one band. The most common XbaI-PFGE profile STYM1 (n = 147) was evenly distributed during the study period. Nonetheless, 50 isolates showed a unique XbaI-PFGE profile. The cluster analysis of MLVA types revealed that no particular clustering emerged between the STM isolates. The majority of the isolates had MLVA type 3-16-NA-NA-0311 but also 89 unique MLVA types were detected (Fig. 2). The most common feature among the Finnish STM isolates was the lack of loci STTR6 and STTR10p, both loci were absent in 70 % of the isolates (260 isolates).

### Concordance of MLVA with phage typing and PFGE

To assess the concordance between the three subtyping methods, adjusted Wallace (AW) and adjusted Rand (AR) coefficients were calculated (Table 6). A poor directional correlation between 5-loci MLVA and XbaI-PFGE and phage typing was found. The probability of two isolates that have the same MLVA type to share the same XbaI-PFGE profile was only 30 % meaning that neither phage type nor PFGE profile could be predicted directly from the MLVA type. According to the AW and AR coefficients, the highest concordance was between XbaI-PFGE and phage typing (AW = 0.833; AR = 0.627).

**Table 6:** Concordance between the typing methods presented by adjusted Wallace (AW) and adjusted Rand (AR) coefficient

	PT	Xbal-PFGE	5-loci MLVA
PT		AW = 0.503	AW = 0.245
Xbal-PFGE	AW = 0.833, AR = 0.627		AW = 0.247
5-loci MLVA	AW = 0.537, AR = 0.336	AW = 0.327, AR = 0.281	

## Discussion

STM is the most frequently isolated *Salmonella* serotype from the Finns who suffer from salmonellosis and have not travelled abroad prior to their symptoms [3]. Thus, accurate surveillance of STYM is of public health importance. Since the 1960s, STM has caused the majority of the domestic salmonellosis in Finland (personal communication, Anja Siitonen, THL). *Salmonella* isolates, however, are rare in Finnish production animals (cattle, poultry and swine) and only about 1 % of the animals tested are salmonella positive [32]. In addition, the phage types circulating among the animals are well known [32, 33]. For example, STM DT1 which was the most common cause of domestic human salmonella infections in this study, is endemic in Finland and isolates with this phage type have been isolated from production animals (poultry and cattle) and wild animals since the 1980s [3, 33, 34]. Phage types DT40 and U277 have been isolated from domestic turkey, wild birds, hedgehogs and regularly from human infections as well [32].

More than 60 % of all the clinical isolates and almost 75 % of all the DT1 isolates were susceptible to all 12 antimicrobials tested in this study. Similar susceptibility results were observed during previous years as well [35]. This could result from moderate usage of antibiotics in Finnish animal husbandry [36]. Nonetheless, 11 % of our isolates were found to be MDR including nine different phage types (DT104, DT104B and DT193 being the most common). Several studies have shown MDR to be more common among certain STM phage types *e.g.* in DT104 [37, 38]. Because MDR *Salmonella* is rare among domestic production animals, the occurrence of MDR isolates among Finnish patients could indicate that at least some of the domestically acquired infections might actually be due to imported foods sold in supermarkets and restaurants [33]. According to a study in Finland, the risk of receiving *Salmonella* from imported beef or beef products was close to 1 % [39]. On the other hand, in a recent source attribution study in Sweden it was estimated that almost 6.5 % of the domestic salmonella infections are due to imported foods [40]. Alternatively, some of the infections could be secondary infections from patients, who have contracted their salmonella infection abroad. Furthermore, about 5 % of the domestic isolates had decreased sensitivity to ciprofloxacin, defined as MIC ≥ 0.125mg/L, which has in previous studies been associated with travelling abroad [41].

Discriminatory power of a method, defined as the ability to distinguish between unrelated isolates, is commonly obtained by calculation of Simpson’s diversity index. In order to get a more objective measure of the subtyping methods used, two different indices, Simpson’s and Shannon’s, were calculated. Both diversity indices provided similar degree of diversity and Shannon’s diversity index (J) also indicated that the different PTs, XbaI-PFGE profiles and MLVA types were equally distributed in the STM population during the study period. In addition, comparable discriminatory power was evidenced for both methods (both DI > 0.8). Sporadic domestic STM isolates showed only limited amount of diversity. The studied isolates showed a genetic similarity of more than 70 % by XbaI-PFGE. In MLVA, absence of two loci was characteristic for domestic STM isolates as 71 % of the isolates lacked STTR6 and 77 % were missing STTR10p loci. Despite of missing loci, 111 distinct MLVA types were observed. According to other studies, MLVA was highly discriminatory (DI > 0.9) for STM [30, 42]. Low diversity observed, especially among the domestic DT1, might result from factors such as geographical isolation, endemic nature of the isolates and lacking of two loci (STTR6 and STTR10pl) in Finnish STM, rather than from the low discriminatory ability of MLVA method as such. This is supported by the fact that the PFGE typing showed low diversity as well. The DT101 isolates in New Zealand [30] and the STM isolates in Malaysia [43] have also been shown to lack STTR6 and STTR10p loci.

Based on the results of this study, The National *Salmonella* Reference Laboratory at THL has since January 2014 used the 5-loci MLVA as the primary genotyping method for STYM isolates and XbaI-PFGE as the secondary method for epidemiological investigations. Despite the comparable degree of discriminatory power for XbaI-PFGE and MLVA, the latter was more cost-effective, faster to perform and above all, easier to interpret and apply in international comparison. However, in order to interpret 5-loci MLVA results appropriately in outbreak situation, more information on VNTR mutation rate and their stability is needed. Some studies have been conducted to determine the VNTR stability. For example, the influence of multiple freeze-thaw cycles on single-colony isolates and the effects of external stress to bacterial genotype have been studied. A study performed with *Escherichia coli* O157:H7 revealed that certain stress factors (temperature, UV-light and starvation) can affect the mutation rate of multiple tandem repeats in one locus more [44]. Another stability study revealed that changes were observed mainly in MLVA loci STTR6, STTR10 and STTR5, but a single change was also detected in STTR9 [38]. In conclusion, the majority of the stability studies agree that the order of instability among STM loci is following STTR6 > STTR10 > STTR5 > STTR9 > STTR3. Nevertheless, as with any typing method, the results obtained with MLVA must be viewed in conjunction with the epidemiological data. Importantly, international criteria for cluster and outbreak investigations should be agreed on.

## Conclusions

In Finland, domestically acquired infections caused by salmonella serotype Typhimurium account for approximately 30 % of all domestic salmonella infections. In this study, the three most common phage types, XbaI-PFGE and 5-loci MLVA profiles comprised 65, 56 and 49 % of the 375 sporadic STM isolates, respectively. More than 60 % of the isolates were susceptible to all 12 antimicrobials tested. For the most common phage types, the 5-loci MLVA had better discriminatory power than XbaI-PFGE. Furthermore, in a daily use, MLVA is simple, fast, robust and cost-effective technique for subtyping STM isolates. The MLVA results are easy to interpret and reproduce, and importantly, the protocol is internationally harmonised allowing the comparison of the results between laboratories. In Finland, MLVA is used as routine subtyping method for surveillance and cluster detection of domestic STM isolates. Nonetheless, phenotyping methods (phage typing and antimicrobial susceptibility testing) are useful for outbreak detection, especially when analyzing isolates with a common MLVA type.

## Methods

### Bacterial isolates

*Salmonella* Typhimurium isolates were received from the Finnish routine clinical microbiology laboratories that are obliged to send all their *Salmonella* isolates to THL based on the Finnish Communicable Disease Act. During the 5-year period (November 2007 to October 2012), all of the 455 STM isolates that had antigen structure 4,5,12:i:1,2 and that were associated with domestically acquired salmonelloses were subtyped at the Bacterial Infections Unit, National Institute for Health and Welfare (THL), Finland. In this study, a *Salmonella* isolate was called domestic if it was isolated from a person who had no travel history in one week prior to his or her symptoms. In case of multiple STM findings in the person, the first finding was chosen for this study. Eighteen percent (80/455) of the original isolates were associated with a known STM infection epidemic or a cluster or were family-related (based on the same family name) and were therefore excluded from the study, leaving 375 sporadic isolates for the analyses.

### Phenotypic characterization

All the isolates were serotyped by slide agglutination according to the White-Kauffmann-Le Minor scheme [45]. The definite phage typing of STM isolates was performed using a standard set of 38 typing phages received from the Public health England [46, 47]. The isolates showing a pattern that did not conform to any recognized phage patterns were designated as “reacts but did not conform” (RDNC). Furthermore, the isolates belonging to RDNC were additionally grouped based on their phage reactions if more than one isolate with similar phage reactions (difference of 1−3 reactions) were detected (RDNC1-5). Isolates that did not react with any of the typing phages were designated as “not typeable” (NT). The antimicrobial susceptibility to 12 antimicrobials was determined by the agar diffusion method on Müller-Hinton II agar for ampicillin (A) (10 μg), chloramphenicol (C) (30 μg), streptomycin (S) (10 μg), sulphonamide (Su) (300 μg), tetracycline (T) (30 μg), ciprofloxacin (Cp) (5 μg), trimethoprim (Tm) (5 μg), gentamicin (G) (10 μg), nalidixic acid (Nx) (30 μg), cefotaxime (Ct) (5 μg), mecillinam (M) (10 μg) and imipenem (I) (10 μg). During 2007–2010, the protocols and clinical breakpoints of the Clinical and Laboratory Standards Institute (CLSI) [48] and during 2011–2012 those of the European Committee on Antimicrobial Susceptibility Testing (EUCAST) (http://www.eucast.org/) were applied. Minimal inhibitory concentration (MIC) for ciprofloxacin (from 0.002 to 32 mg/L) was detected by E-Test (Biomérieux, Marcy l’Étoile, France) for the isolates that were resistant (R) or intermediate resistant (I) to Nx. MIC breakpoint ≤1 mg/L was interpreted as susceptible [48]. Multidrug resistance (MDR) was defined as resistance to four or more antimicrobials.

### Genotypic characterization of the isolates

#### PFGE

For PFGE typing, the isolates were cultivated overnight on trypticase soy agar (TSA) plates, and PFGE was performed according to the internationally standardized PulseNet protocol [49] using 15 U XbaI restriction enzyme (Roche, Basel, Switzerland) for each plug. XbaI-digested *S*. Braenderup (H9812) served as a DNA size marker. Banding patterns were analysed using BioNumerics v.6.6 (AppliedMaths, Kortrijk, Belgium). The bands within a size range of 33 kb and 1135 kb were included in the analysis, and isolates differing even in one banding position or in thickness of the band were assigned as different PFGE types. An unweighted pair group method with arithmetic mean (UPGMA) dendrogram was constructed using a Dice coefficient.

#### MLVA

The MLVA was performed as described in [27, 31, 50] with some modifications (Table 1). Isolates were grown overnight on nutrient agar plates, and 1-2 colonies were suspended into 500 μl sterile water and boiled for 5 min. After a quick centrifugation, 1 μl of the supernatant was used as template in each PCR reaction. For ABI3730xl (G5 filter) suitable fluorescence-labelled forward primers (STTR3-F-NED, STTR5-F-NED, STTR6-F-FAM, STTR9-F-FAM and STTR10F-PET) were ordered from Applied Biosystems (CA, USA) and the unlabelled reverse primers from Oligomer (Espoo, Finland). A 5-plex PCR reaction was performed with a Qiagen multiplex kit (Hilden, Germany) in a total volume of 25 μl and included 2.50 pmol of primers STTR3-F, STTR3-R, STTR6-F, and STTR6-R and 1.25 pmol of primers STTR5-F, STTR5-R, STTR9-F, STTR9-R, STTR10pl-F, and STTR10pl-R. Amplification was performed with a Dyad Peltier thermal cycler (Bio-Rad, Hercules, USA), starting with 15 min at 95 °C, followed by 30 cycles of 30 s at 94 °C, 90 s at 60 °C, and 90 s at 72 °C and ending with an extension step for 10 min at 72 °C. The PCR products were diluted in a ratio of 1:85 in sterile ddH_2_O. Total of 10 μl of formamide/size standard mixture containing 2 μl of internal size standard GeneScan™ 600 LIZ ® (Applied Biosystem, Forter City, CA, USA) and 1 ml Hi-Di™ formamide (Applied Biosystems, Foster City, CA, USA) was applied to 96-well plates. Then, 1 μl of diluted PCR product (1:85) was added into each well. The PCR products were separated with an ABI3730xl automated DNA analyzer (Applied Biosystems, CA, USA). The size and dye label associated with each amplicon were determined using Peak Scanner v.1.0 software (Applied Biosystems, Foster City, CA, USA). Initially, a set of 33 calibration STM isolates and a correction table provided by Dr. Eva Møller Nielsen, Statens Serum Institute, Denmark, were used to calibrate the method and to normalise the raw data for the correct determination of the number of repeat units in each locus. Based on the fragment length, repeat numbers were assigned for each isolate by using arbitrary numbers [31]. Unique allelic combinations were assigned as a separate MLVA type, and all MLVA types were reported as repeat number values in the following order: STTR9-STTR5-STTR6-STTR10-STTR3. A null amplification product was considered a distinct allele after confirmation by a repeated single PCR assay and markedas -2 in the Bionumerics. MLVA results were stored at Bionumerics v.6.6 (Applied Maths, Kortrijk, Belgium). A UPGMA dendrogram was constructed using the categorical values coefficient.

#### Analysis

Discriminatory power of phage typing, XbaI-PFGE and 5-loci MLVA were assessed using Simpson’s index of diversity (DI) and the Shannon’s diversity index (H’), which are indicator of species richness (*i.e.* number of subtypes), and equitability (J), which is a measure of the evenness of subtype distribution, *via* the online tool (www.comparingpartitions.info). Additionally, the diversity index was assigned for each MLVA loci (http://www.hpa-bioinformatics.org.uk/cgi-bin/DICI/DICI.pl). Simpson’s DIs ranges from zero (no diversity) to one (high diversity). Confidence intervals were calculated for Simpson’s DIs as described earlier [51]. The *χ*^2^ test was used to determine the significance of the difference between typing methods, and a p*-*value of <0.05 was considered indicative of a significant difference. The concordance of different typing methods was determined using adjusted Rand (AR) and adjusted Wallace (AW) coefficients (www.comparingpartitions.info). AR is a coefficient suitable for quantitative evaluation of the concordance between different microbial typing methods. AR values range from zero to one (global congruence of typing method is high). Wallace’s coefficient indicates the probability at which two isolates of the same type are also classified as the same by another method.
